# Supplementary material for: Characterisation of miRNA Expression in Dental Pulp Cells during Epigenetically-Driven Reparative Processes
Source: Int J Mol Sci. 2023 May 11;24(10):8631. doi: 10.3390/ijms24108631 (PMC10218409; doi:10.3390/ijms24108631)
Supplement: Supplementary file 1 [file ijms-24-08631-s001.zip › ijms-2363607-supplementary.pdf]

Ind vs NInd			
**miRNA**	***p*-value**	**Fold change**	**Regulation**
**rno-miR-770-3p**	0.0495	-5.3917	down
**rno-miR-346**	0.0463	-5.0284	down
**rno-miR-201-5p**	0.0495	5	up
**rno-miR-598-5p**	0.0495	-4.7671	down
**rno-miR-375-3p**	0.0495	4.5716	up
**rno-miR-377-5p**	0.0495	-3.1195	down
**rno-miR-3558-5p**	0.0495	3.0575	up
**rno-miR-412-5p**	0.0495	-3.053	down
**rno-miR-376a-3p**	0.0495	-2.934	down
**rno-miR-147**	0.0495	2.8676	up

Table S1. miRNAs with the largest fold change in expression in Ind compared with NInd at day 4 as determined by a Mann-Whitney U test. All data analysis carried out on three independent replicates. Fold change cut-off >1.5, $p \leq 0.05$.

Ind+S vs NInd			
**miRNA**	***q*-value**	**Fold change**	**Regulation**
**rno-miR-3553**	0.0466	8.481	up
**rno-miR-410-5p**	0.0467	8	up
**rno-miR-182**	0.0466	3.6954	up
**rno-miR-377-3p**	0.0466	3.3264	up
**rno-miR-1247-3p**	0.0466	-3.2453	down
**rno-miR-708-3p**	0.0454	-3.093	down
**rno-miR-758-3p**	0.0496	3.0928	up
**rno-miR-10a-5p**	0.0466	3.011	up
**rno-miR-543-3p**	0.0466	2.932	up
**rno-miR-540-3p**	0.0466	2.7322	up

Table S2. miRNAs with the largest fold change in expression in Ind+S compared with NInd at day 4 as determined by a moderated t-test in combination with Storey's bootstrapping method. All data analysis carried out on three independent replicates. Fold change cut-off >1.5, $q \leq 0.05$.

**Ind+A vs NInd**			
**miRNA**	***q*-value**	**Fold change**	**Regulation**
**rno-miR-881-3p**	5.56E-04	603.906	up
**rno-miR-741-3p**	2.53E-04	249.7982	up
**rno-miR-871-3p**	2.53E-04	213.5208	up
**rno-miR-871-5p**	0.001	199.2512	up
**rno-miR-743b-3p**	0.001	194.4895	up
**rno-miR-743a-3p**	2.53E-04	178.295	up
**rno-miR-463-5p**	0.001	161.3718	up
**rno-miR-743b-5p**	4.06E-04	98.8655	up
**rno-miR-3580-3p**	0.002	51.3771	up
**rno-miR-471-5p**	2.53E-04	36.765	up

Table S3. miRNAs with the largest fold change in expression in Ind+A compared with NInd at day 4 as determined by moderated t-test in combination with Storey's bootstrapping. All data analysis carried out on three independent replicates. Fold change cut-off >1.5, $q \leq 0.05$.

**Ind+S vs Ind**			
**miRNA**	***q*-value**	**Fold change**	**Regulation**
**rno-miR-346**	0.0144	9.0318	up
**rno-miR-3553**	0.0231	7.1433	up
**rno-miR-412-5p**	0.0231	7.0871	up
**rno-miR-410-5p**	0.0329	6.8276	up
**rno-miR-377-5p**	0.0326	6.2164	up
**rno-miR-183-5p**	0.0311	5.6106	up
**rno-miR-770-3p**	0.0276	5.4077	up
**rno-miR-377-3p**	0.0156	4.9179	up
**rno-miR-6331**	0.0184	4.6497	up
**rno-miR-667-5p**	0.0215	4.5881	up

Table S4. miRNAs with the largest fold change in expression in Ind+S compared with Ind at day 4 as determined by moderated t-test in combination with Storey's bootstrapping. All data analysis carried out on three independent replicates. Fold change cut-off >1.5, $q \leq 0.05$.

**Ind+A vs Ind**			
**miRNA**	***q*-value**	**Fold change**	**Regulation**
**rno-miR-881-3p**	0.0066	273.9668	up
**rno-miR-743b-3p**	0.002	240.7617	up
**rno-miR-871-3p**	0.003	214.3537	up
**rno-miR-743a-3p**	1.94E-04	191.8932	up
**rno-miR-463-5p**	0.0015	175.1376	up
**rno-miR-741-3p**	0.0015	172.5662	up
**rno-miR-871-5p**	0.003	153.8468	up
**rno-miR-743b-5p**	0.001	86.0507	up
**rno-miR-3580-3p**	0.0023	51.3771	up
**rno-miR-471-5p**	1.94E-04	36.765	up

Table S5. miRNAs with the largest fold change in expression in Ind+A compared with Ind at day 4 as determined by moderated t-test in combination with Storey's bootstrapping. All data analysis carried out on three independent replicates. Fold change cut-off >1.5, $p \leq 0.05$.

miRNA	Fold change				Notes/reason for selection	Reference	% Sequence similarity with human ortholog
	Ind+S vs NInd	Ind+A vs NInd	Ind+S vs Ind	Ind+A vs Ind			
**rno-miR-200b-3p**	-	2.598	-	1.559	Differentially regulated in Ind+A compared with NInd and Ind		95.70
					Controlled by DNMT3A	Zhao et al., 2018	
**rno-miR-205**	-2.06	3.11	-	3.98	Inhibition of miR-205 enhances osteogenic abilities by upregulating *Bsp* and *Opn*	Hu et al., 2015	95.6%
					Is one of many *Runx2*-targeting miRNAs suggested to be involved in osteogenesis and chondrogenesis	Zhang, et al., 2011	
**rno-miR-221-5p***	1.732	1.667	-	-	Differentially regulated in two of three groups		95.70
					Inhibition of microRNA-221-5p induces osteogenic differentiation of BMMSCs via upregulation of *SMAD3*	Fan et al., 2019	

miRNA	Fold change				Notes/reason for selection	Reference	% Sequence similarity with human ortholog
	Ind+S vs NInd	Ind+A vs NInd	Ind+S vs Ind	Ind+A vs Ind			
**rno-miR-182**	3.695	2.056	2.907	-	Downregulated miR-182-5p promotes the proliferation and differentiation of osteoblasts in osteoporotic rats through Rap1-MAPK signalling	Pan et al., 2018	92
**rno-miR-346**	-	-	9.032	-	Highest differentially regulated in Ind+S vs Ind		87
**rno-miR-881-3p**	-	603.91	-	273.97	Highest differentially regulated in Ind+A vs Ind		N/A

Table S6. Six miRNAs chosen for validation using qRT-PCR. The fold change of each miRNA in each of the 5 experimental pairings as determined by A moderated t-test combined with Storey's bootstrapping method is shown, as well as the percentage sequence identity each miRNA shares with its human ortholog. rno-miR-881-3p does not have a known human ortholog. miR-221-5p was not found to be differentially regulated in any experimental group when using the moderated t-test, however the less stringent Mann-Whitney U test determined it to be differentially expression in Ind+S and Ind+A compared with Ind.
